# Evaluation of Biomarkers in Active Alzheimer's Disease Intervention Clinical Trials – The Biomarker Observatory

**DOI:** 10.1002/alz70856_105220

**Published:** 2026-01-11

**Authors:** Amanda M. Leisgang Osse, Yadi Zhou, Andrew Hooyman, Ayan Sengupta, Jorge Fonseca, Jefferson W Kinney, Feixiong Cheng, Jeffrey L. Cummings

**Affiliations:** ^1^ University of Nevada Las Vegas, Las Vegas, NV, USA; ^2^ Chambers‐Grundy Center for Transformative Neuroscience, Las Vegas, NV, USA; ^3^ Cleveland Clinic, Cleveland, OH, USA; ^4^ Arizona State University, Tempe, AZ, USA; ^5^ Kirk Kerkorian School of Medicine, University of Nevada Las Vegas, Las Vegas, NV, USA

## Abstract

**Background:**

Biomarkers are critically important for Alzheimer's Disease (AD) drug development. They have a role in patient diagnosis and trial eligibility, pharmacodynamic changes, safety, monitoring, disease modification, and target engagement. In AD clinical trials, biomarkers are vital in determining the treatment efficacy, reliability, and safety.

**Method:**

We created the Biomarker Observatory (BMO) to provide curated information on AD biomarkers for drug developers and clinical trialists. From the BMO, our aim is to explore and present the biomarkers that are currently being used in active AD intervention trials. We extract information from clinicaltrials.gov and annotate the eligibility, primary, secondary, and other biomarkers from each trial. Together with information from the Clinical Trial Observatory, we explore the imaging, fluid, and digital biomarkers being measured in active AD clinical trials, the trial characteristics, and influence of biomarkers on trials.

**Result:**

For active trials in the year 2024 (January 1 to December 30, 2024), we found that 57% of the clinical trials used a biomarker as an eligibility criterion for patient participation. Imaging was the most common type of marker, with magnetic resonance imaging (MRI) and amyloid positron emission tomography (PET) most used as eligibility and primary outcome biomarkers. Fluid biomarkers (CSF, blood, plasma, or serum) were used in 28% of AD clinical trials as eligibility biomarkers and in 12% of trials as a primary outcome measure. Seventeen AD clinical trials measured amyloid beta protein (Aβ) in fluid samples as a criterion for eligibility and 17 evaluated hyperphosphorylated tau (*p*‐tau). As a primary outcome measure, 2 trials measured Aβ in fluid samples and 7 investigated *p*‐tau levels. Total tau and neurofilament light chain (NfL) were included as primary outcome measures in 2 and 3 trials, respectively.

**Conclusion:**

Our data demonstrate how imaging, fluid, and digital biomarkers are used in current AD clinical trials and the impact they have on trial design and outcomes. We provide current biomarker information to advance AD drug development, aiming to increase trial success rates and reducing the time it takes for new AD therapeutics to reach patients.

